# Virtual reality and non-invasive brain stimulation for rehabilitation applications: a systematic review

**DOI:** 10.1186/s12984-020-00780-5

**Published:** 2020-10-31

**Authors:** Raymundo Cassani, Guilherme S. Novak, Tiago H. Falk, Alcyr A. Oliveira

**Affiliations:** 1 Institut National de La Recherche Scientique (INRS-EMT), University of Quebec, 800 rue de la Gauchetière O, Montreal, QC H5A-1K6 Canada; 2 Department of Psychology, Federal University of Health Sciences of Porto Alegre, Rua Sarmento Leite, 245, Porto Alegre, CEP 90.050-170 Brazil

**Keywords:** Virtual reality, Non-invasive brain stimulation, Transcranial direct current stimulation, Transcranial magnetic stimulation, Rehabilitation

## Abstract

The present article reports the results of a systematic review on the potential benefits of the combined use of virtual reality (VR) and non-invasive brain stimulation (NIBS) as a novel approach for rehabilitation. VR and NIBS are two rehabilitation techniques that have been consistently explored by health professionals, and in recent years there is strong evidence of the therapeutic benefits of their combined use. In this work, we reviewed research articles that report the combined use of VR and two common NIBS techniques, namely transcranial direct current stimulation (tDCS) and transcranial magnetic stimulation (TMS). Relevant queries to six major bibliographic databases were performed to retrieve original research articles that reported the use of the combination VR-NIBS for rehabilitation applications. A total of 16 articles were identified and reviewed. The reviewed studies have significant differences in the goals, materials, methods, and outcomes. These differences are likely caused by the lack of guidelines and best practices on how to combine VR and NIBS techniques. Five therapeutic applications were identified: stroke, neuropathic pain, cerebral palsy, phobia and post-traumatic stress disorder, and multiple sclerosis rehabilitation. The majority of the reviewed studies reported positive effects of the use of VR-NIBS. However, further research is still needed to validate existing results on larger sample sizes and across different clinical conditions. For these reasons, in this review recommendations for future studies exploring the combined use of VR and NIBS are presented to facilitate the comparison among works.

## Background

Virtual reality (VR) is a medium that is typically composed of an interactive computer simulation which detects the actions and position of the subject, additionally, it replaces or augments the feedback (e.g., visual, auditory, haptic) to the user, providing a sensation of presence in the simulation [1–3]. The last decade has witnessed a drastic improvement in computer graphics and computational power, which in turn, have paved the road to more realistic virtual- and augmented-reality systems and experiences, with applications in entertainment, gaming, e-commerce, architecture, interior design, manufacture, education, health and medicine [4]. Regarding rehabilitation, there is strong evidence supporting the use of VR therapy [5–7] in the treatment of pain, phobias, post-traumatic stress disorder (PTSD) [5], eating disorders [8], mental disorders, such as anxiety, schizophrenia and autism [9], and chemical abuse [10]. Moreover, VR has proven to be an important tool for exposure therapy [11]. Interestingly, a recent review on the medical literature has revealed that no reports of photosensitive epilepsy evoked by the use of VR headset [12].

Non-invasive brain stimulation (NIBS) techniques, in turn, have been consistently studied in the treatment of neuropsychiatric diseases as methods to modify or modulate the cortical excitability [13], and plasticity in the cerebral cortex [13, 14]. The two most common techniques used for NIBS are transcranial magnetic stimulation (TMS) and transcranial electrical stimulation (tES). As the name suggests, TMS uses magnetic fields, more specifically, their rapid change to induce a short pulse of electric current on the cortex, which in turn generates action potentials with a depth up to 5 cm. The application of the magnetic fields is carried out by a magnetic coil that is placed near the scalp over the cortical region of interest. TMS can be used in different ways, as single-pulse, repetitive TMS pulses (rTMS) [15, 16], or intermittent theta burst stimulation (iTBS) when magnetic pulses are intermittently applied in a specific burst [17]. NIBS with TMS have been used for the treatment of depression and schizophrenia [18, 19], pain [18], obsessive–compulsive disorder, Parkinson’s disease, epilepsy, task-related dystonia, and tic disorders [19]. On the other hand, tES relies on the passage of a weak electric current between electrodes placed on the scalp, thus stimulating the brain tissues between the electrodes. Depending on the type of electric current that is used, tES can be further divided into transcranial direct current stimulation (tDCS), alternating current stimulation (tACS), and random noise stimulation (tRNS) [20]; with tDCS being the most common type [14]. As tDCS possesses polarity, it can be anodal or cathodal, thus depolarizing or hyperpolarizing the resting membrane potential, respectively. This is reflected as an increase or decrease on the cortical excitability, respectively [20, 21]. Reported therapeutic applications of tDCS include treatment of pain, Parkinson’s disease, Alzheimer’s disease, motor disorders, stroke, aphasia, multiple sclerosis, epilepsy, depression, schizophrenia, and substance abuse [13]. In studies using TMS and tES, a “sham” (placebo) condition is often used as control. For tES, the sham condition consists in administering real tES to the subject during only few seconds at the beginning of the experiment to mimic the perception and experience of real stimulation. In TMS, there are two approaches for the sham condition: in one approach a TMS coil is placed in a position and orientation that evokes the somatosensory effects of real TMS but brain stimulation is absent; on the other approach, a sham TMS coil that resembles a regular TMS coil but is equipped with a magnetic shield that attenuates the magnetic field, additionally, electrical stimulation can be used to replicate the somatosensory effects of real TMS [22].

Recently, evidence has emerged showing promising therapeutic applications for the combined use of VR and tES (more specifically tDCS) [23–26], as well as VR and TMS [27–29], with outcomes not achievable by using either technique individually. Despite the reported promising results, the literature still lacks a systematic review covering the reported methods, outcomes, and potential therapeutic applications in neurological rehabilitation, on the combination of VR and NIBS techniques. This review aims to fill this gap, by reporting, comparing, and discussing studies that used VR-NIBS therapy for rehabilitation applications. Moreover, this review provides recommendations for future studies in the field to facilitate their development and comparison.

## Methods

A survey on English peer-reviewed journal articles that described the combined use of VR and NIBS for therapeutic applications was performed. Six major bibliographic databases, PubMed, Science Direct, Web of Science, Scopus, Cochrane Library and Google Scholar were queried, with the last query performed in July 2020. The search terms that were used included:

1. “virtual reality”
2. “transcranial magnetic stimulation”
3. “transcranial direct current stimulation”
4. “transcranial alternating current stimulation”
5. “transcranial random noise stimulation”
6. “brain stimulation”.

These search terms were combined as 1 and (2 or 3 or 4 or 5 or 6). All abbreviations were also searched, i.e., “VR”, “TMS”, “tDCS”, “tACS”, “tRNS”, and “NIBS”. We refined the results by limiting the results for search terms found only in titles or abstracts. This step was taken to ensure that our data did not include studies outside the scope of this review. The selection criteria included the combined use of VR and NIBS described in clinical trials and case studies (study protocols were excluded) with no restrictions on publication date. We excluded material that did not include the use of both techniques as a combined treatment for the same participants, e.g., works where TMS mapping was used to evaluate the results of VR therapy sessions. Lastly, another criterion for exclusion was the use of VR systems that were not interactive, e.g., works where the VR element of the system was comprised by the presentation of videos or images. The selection process was performed by at least two independent researchers, and in case of disagreement the final decision was reached by after a discussion between the two researchers.

To facilitate the comparison and discussion over the diverse methodologies presented in the reviewed articles, items that are vital to characterize the reported study were extracted for each article. For this purpose, a data extraction spreadsheet was designed. For each article, 23 items were extracted. These items were grouped in four categories: study rationale, study design, experimental protocols, and reported outcomes. The category *study rationale* comprises the therapeutic application explored in the study, and the main study goal regarding the VR-NIBS combination. The *study design* category encompasses study characteristics such as population, experimental conditions, and blinding approach. The *experimental protocols* category describes the VR protocol, the NIBS protocol and how these were combined. Lastly, the *reported outcomes* category describes the methods and metrics used to evaluate the effects of the VR-NIBS protocol, the main conclusions regarding the combined use of VR and NIBS, and the reported limitations. These four categories, their respective items, and descriptions are presented in Table 1.

**Table 1:** Extracted data items from each article

Category (# of items)	Data items	Description
Study rationale (2)	Therapeutic application	Application, i.e., rehabilitation, mental disorder
	Main study goal	Purpose for combining VR and NIBS
Study design (3)	Study population	Description of the population in the study
	Experimental conditions	Description of experimental conditions in the study
	Blinding approach	Blinding method used in the study
Experimental protocols (15)	VR protocol (6)	Type of VR system, description, viewpoint, duration, hardware, and software
	NIBS protocol (6)	Type of NIBS, subtype of NIBS, description, duration, intensity, and hardware
	VR-NIBS protocol (3)	Temporal relation, duration, and number of sessions
Reported outcomes (3)	Evaluation methods	Methods to assess the obtained outcomes
	Main conclusions	Conclusions about the combined use of VR and NIBS
	Reported limitations	Limitations reported in the study

## Results and discussion

A total of 447 articles were retrieved from the databases and after removing duplicates, 304 articles were retained. By analyzing the title and abstract, 274 articles were excluded. A further refinement resulted in 17 articles rejected because they were outside of the scope of this review. As such, after these steps, 13 articles were considered relevant for the current review. Additionally, 3 articles were included based on analysis of the references cited in the originally selected 13 articles, thus totalling 16 articles included in our analysis. The publication date of the reviewed articles ranged from 2010 to 2020, which was not surprising as the use of the VR-NIBS combination in therapeutic applications is recent. A total of 23 items (presented in Table 1) were extracted from each of the reviewed articles. The following subsections discuss the similarities and differences for these items across the reviewed articles. Despite the diverse NIBS techniques, presented in “Background” section, only TMS and tDCS were reported as NIBS techniques in the reviewed articles, this is further discussed in “NIBS protocol” section.

### Category: study rationale

#### Therapeutic application

According to the main reported therapeutic application, the reviewed articles were grouped into five major categories. Studies which explored combination of the VR and NIBS for therapy in (i) stroke rehabilitation, (ii) phobia and PTSD, (iii) cerebral palsy, (iv) neuropathic pain, and (v) multiple sclerosis. The most commonly reported application was stroke rehabilitation being reported in seven of the reviewed articles, interestingly all these articles were focused on therapy of the upper limb (UL).

#### Main study goal

Although the reported goals are specific for each study, it is possible to group them into three major categories depending on the reported hypotheses for the combined use of VR and NIBS. More specifically, these categories are: (i) studies that evaluated the effects of VR- and NIBS-based therapies separately and jointly (i.e., VR-NIBS); (ii) studies that evaluated the effects of VR-based therapy, and the addition of NIBS to it, i.e., VR-NIBS; and (iii) studies that did not evaluate VR- nor NIBS-based separately, but only jointly VR-NIBS therapy. A brief description of the main goal of each the reviewed articles is presented in Table 2, alongside the category of this main goal, and the reported therapeutic application.

**Table 2:** Study rationale: therapeutic application and main study goal

Therapeutic application	Main goal category	Article	Main goal description. The article mainly studies:
Stroke rehabilitation	(i)	[25] [30]	The effects of NIBS, VR and its combination on therapy for upper limb training in patients with subacute stroke. The effects of combining NIBS with VR-based motor skill training in patients with subacute stroke
	(ii)	[31]	The effect of adding NIBS to VR therapy, for upper limb training in unilateral stroke
		[29]	Whether combining NIBS with VR training could improve upper limb function in subacute stroke patients
		[28]	The effects of adding NIBS to a VR-BCI therapy for motor recovery after stroke
		[32]	The effects of adding NIBS in VR therapy to improve upper limb motor function after stroke
	(iii)	[33]	The effects of NIBS-VR paradigm for upper limb rehabilitation in a stroke survivor with severe hemiparesis
Phobia and PTSD	(ii)	[34] [35]	The effects on acute anxiety of adding NIBS to a VR experience for patients with spider phobia The impact on emotion regulation of adding NIBS to a VR experience for patients with spider phobia
		[36]	The use of NIBS during VR experience to reduce psychophysiological arousal and symptoms in veterans with PTSD
Cerebral palsy	(ii)	[37]	The effects of a single session of NIBS combined with VR training on functional mobility in children with cerebral palsy
		[26]	The effects of a single session of NIBS combined with VR training on the balance of children with cerebral palsy
		[38]	The effects of multiple sessions of NIBS combined with VR training on the balance of children with cerebral palsy
Neuropathic pain	(i)	[39]	The analgesic effect of using NIBS on the motor cortex, and VR techniques, applied isolated or combined
	(iii)	[40]	The effects on pain relief of a NIBS-VR intervention, to improve neuropathic pain in patients with severe spinal cord injury
Multiple sclerosis	(ii)	[41]	The effects of VR combined NIBS on balance, fatigue, and quality of life in a patient with multiple sclerosis

The main study goal was grouped according to three categories, studies that evaluated: (i) studies that evaluated the effects of VR- and NIBS-based therapies separately and jointly (i.e., VR-NIBS); (ii) studies that evaluated the effects of VR-based therapy, and the addition of NIBS to it, i.e., VR-NIBS; and (iii) studies that did not evaluate VR- nor NIBS-based separately, but only jointly VR-NIBS therapy

The most frequent study goal category was to explore the potential benefit of adding a NIBS technique to a previously verified VR-based therapy, category (ii), this was reported in 11 of the 16 reviewed articles, this is because VR-based therapies have been widely explored for treatment of phobias, PTSD [42], and cerebral palsy [43]. Outstandingly, we identify only three studies, [25, 30, 39], that evaluated the effects of VR and NIBS separately and jointly, main goal category (i). These works offer a richer perspective in the ways that VR and NIBS techniques complement each other. On the other side, in works that only explore the effects of the combined use of VR and NIBS, main goal category (iii), it is not possible to evaluate the contributions of VR and NIBS separately, this is further expanded in “Reported outcomes” section.

### Study design

#### Study population

In most of the studies, 12 studies, the study population was comprised solely by patients. In the remaining studies, [30, 34, 35, 40], the population included patients and healthy participants. The reported number of participants who completed the study protocols greatly varied from 1 up to 108, with an average of 38 participants. Details on the study population study can be found in Table 3.

**Table 3:** Study design: population, conditions, and blinding approach

Article	Study Population	Experimental Conditions
[25]	Patients with impaired unilateral UL motor function due to stroke (n = 59)	Participants were randomly assigned to 3 groups: Occupational therapy + tDCS (n = 19) (A) VR instead of occupational therapy (n = 20) (B) VR therapy + tDCS (n = 20)
[30]	Patients with stroke in the subacute stage (n = 15), and healthy participants (n = 15)	(C) All participants underwent 4 conditions in random order, in different consecutive days: (A) Active wrist exercise (B) VR wrist exercise (C) VR wrist exercise + tDCS (D) tDCS without wrist exercise
[31]	Patients with impaired unilateral UL motor function due to unilateral stroke (n = 20)	Participants were randomly assigned to 2 groups: (A) VR + tDCS (n = 10) (B) VR + sham tDCS (n = 10)
[29]	Patients with hemiplegia after stroke (n = 108)	Participants were randomly assigned to 2 groups: (A) VR + TMS (n = 55) (B) VR + sham TMS (n = 53)
[28]	Patients with impaired motor function due to stroke (n = 3)	Participants were randomly assigned to 2 groups: (A) VR + TMS (n = 2) (B) VR + sham TMS (n = 1)
[33]	Patient with severe left hemiparesis due to stroke (n = 1)	Participant underwent A-B-A conditions: (A) Motor rehabilitation (no VR nor tDCS) (n = 1) (B) Motor rehabilitation + VR + tDCS (n = 1)
[32]	Patients with ischemic stroke (n = 40)	Participants were randomly assigned to 2 groups: (A) VR + tDCS (n = 20) (B) VR + sham tDCS (n = 20)
[34]	Patients with spider phobia (n = 41), and healthy participants (n = 42)	Participants were randomly assigned to 2 groups: (A) VR + TMS (n = 40) (B) VR + sham TMS (n = 43)
[35]	Patients with spider phobia (n = 41), and healthy participants (n = 42)	Participants were randomly assigned to 2 groups: (A) VR + TMS (n = 40) (C) VR + sham TMS (n = 43)
[36]	Patients with PTSD (n = 12)	Participants were randomly assigned to 2 groups: (A) VR + tDCS (n = 6) (B) VR + sham tDCS (n = 6)
[37]	Children patients with cerebral palsy (n = 12)	Participants were randomly assigned to 2 groups: (A) VR + tDCS (n = 6) (B) VR + sham tDCS (n = 6)
[26]	Children patients with cerebral palsy (n = 12)	Participants were randomly assigned to 2 groups: (A) VR + tDCS (n = 6) (B) VR + sham tDCS (n = 6)
[38]	Children patients with cerebral palsy (n = 20)	Participants were randomly assigned to 2 groups: (A) VR + tDCS (n = 10) (B) VR + sham tDCS (n = 10)
[39]	Patients with SCI and NP (n = 39)	Participants were randomly assigned to 4 groups: (A) VR + tDCS (n = 10) (B) tDCS group (n = 10) (C) VR group (n = 9) (D) Placebo group (n = 10)
[40]	Patients with SCI and NP (n = 18), patients with SCI without NP (n = 20), and healthy participants (n = 14)	Only SCI patients with NP underwent: VR + tDCS therapy (n = 18)
[41]	Patient with primary-progressive MS (n = 1)	Participant underwent A-B conditions: (A) VR + tDCS (n = 1) (B) VR + sham tDCS (n = 1)

*MS* multiple sclerosis, *NP* neuropathic pain, *UL* upper limb, *SCI* spinal cord injury

#### Experimental conditions and blinding approach

The set of experimental conditions that were reported in the reviewed articles depended on the main goal of the study (presented in “Main study goal” section). In 12 studies, the participant study population was divided into groups that received different therapies, whereas in the remaining studies, [30, 33, 40, 41], the entire participant population underwent all the different therapies. The reported study population and experimental conditions are presented in Table 3, in this table, the population numbers correspond to number of participants who completed the experimental protocol.

The reviewed studies reported different blinding approaches to prevent both participant and/or experimenter biases [44]. Six studies, [26, 29, 31, 37–39], reported double blinding (patients and therapists); single blinding on the patient side was reported in seven studies [28, 30, 32, 34–36, 41]; and the remaining three studies reported single blinding only in the therapist side.

### Experimental protocols

In this Section, first, the VR and NIBS protocols are presented separately with the purpose of comparison across studies. Later, the combined VR-NIBS protocols are described and discussed.

#### VR protocol

The definition of VR, presented in “Background” section, encompasses a large variety of systems that present a virtual environment (VE) to the subject, i.e., though computer monitors, single screen projectors, rooms which walls are immersive projections, and head-mounted displays (HMD), among others. Nevertheless, the interactive nature of the VR system must be present.

**Fig. 1:**
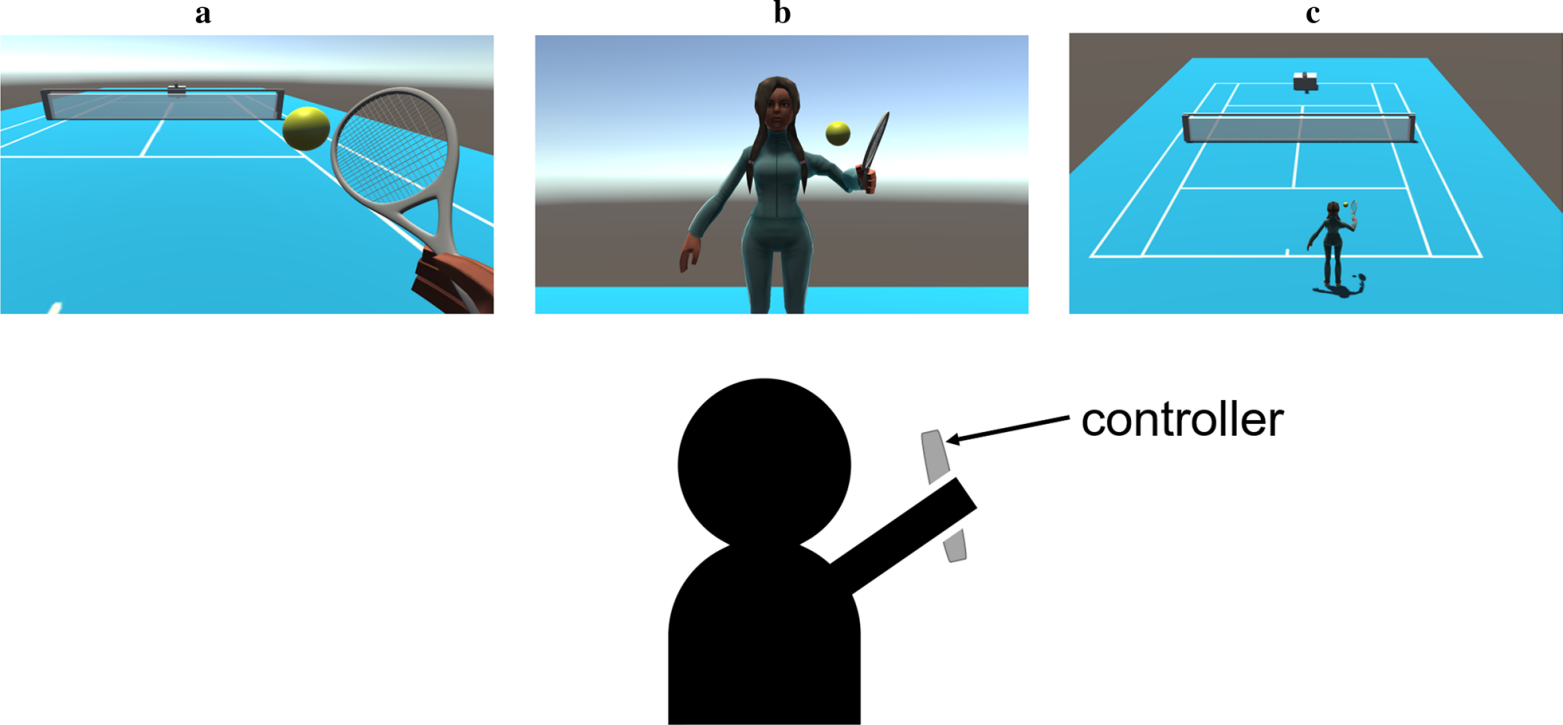
Example of a stationary VR system where the user actions are mapped to the virtual tennis player through a controller. Different viewpoints for the same VE are presented: **a** 1PP, **b** 1PP-mirror and **c** 3PP

In this review, the multiple reported VR systems are grouped into two categories: (i) stationary, and (ii) head-based. This classification is based on the way the VE is presented to the subject, whether it is always present (stationary), or rendered according to the head position of the subject (head-based) [2]. Another relevant aspect for the VR system is the subject’s viewpoint in the VE, which can be either first-person and third-person perspective, 1PP and 3PP respectively. In 1PP, the VE is presented from the point of view of the virtual entity that represents the subject. As its name indicate, in 3PP, the VE is presented as it were seen by a third person, thus the subject can see the entity that represents them in the VE, and the VE. The use of 1PP viewpoint has been proven to better induce a sense of embodiment toward a virtual body, especially in aspects of self-location and ownership, relative to the 3PP approach [45]. Head-based VR systems present 1PP viewpoint, while this is not necessarily true for stationary VR systems. In the reviewed articles a specific 1PP viewpoint was commonly found, in which the subject can see either the entity that represents them or themselves in the VE as if they were reflected in a mirror, we identify this viewpoint as 1PP-mirror. Figure 1 illustrates the 1PP, 1PP-mirror and 3PP viewpoints for the same VE in a stationary VR system.

In the reviewed articles, stationary VR systems were the most commonly used, reported in 13 articles. It is important to note that the 3 articles, [34, 35] and [36], that used head-based VR systems, relied on VR devices that are 10 years or older, thus they may not be appropriate for realistic VR experiences. Regarding the duration of the VR experience, there is not consensus among the review articles, reported duration ranged from 6 to 45 min. To make a fair comparison between VR protocols, for each of the reviewed articles, a brief description of the reported VR protocol, type of VR system, viewpoint, and duration are presented in Table 4. The hardware and software that were used in each of the reviewed articles in provided as Additional file 1: Table S1.

**Table 4:** Characteristics of the VR protocol

VR type	Article	Description	Viewpoint	Duration
Stationary	[25]	A video camera recognized the movements and position of the patient in a green room. In a monitor, the patient can see an image of herself in the VE. The patient interacts with virtual objects with a glove	1PP-mirror	30 min
	[30]	A computerized VR ski game was presented in a computer monitor. The interaction was carried on by a cylinder-like object that was grasped by the subject	1PP	15 min
	[31]	Patients played three Nintendo Wii games on a TV screen. These games provided various types of exercises for the UL, including movements of the shoulder, elbow, wrist, hand, and fingers	Depends on the game	45 min
	[29]	Wearable data gloves with sensors, the patient was seated in a comfortable chair with armrests performing shoulder, elbow, and wrist exercises. A therapist chose the therapy according to the needs and abilities of the patient	3PP	30 min
	[28]	Patients were seated in a comfortable chair and wore 3D glasses in front of a stimulus presentation box, in which they would place their hands to grasp and lift a cup. The box hid the hands of the patient and displayed on screen the VR stimulus which consisted of VR hands aligned with the perception of patient hands	1PP	N/R
	[33]	The VE was presented in a laptop screen and showed both left and right virtual arms. The objective of the task was to pick up apples. For this, the patient had to attempt the reaching movement and look at the apple on the screen. The interaction was carried out with an eye-tracking device and armband capable of measuring EMG and position. Patient was seated in a comfortable chair	1PP	20 min
	[32]	The VE was presented in a large screen and consisted of a game in which the patient had to hit a target object with a virtual ball through the movement of a mechanical handle	N/R	20 min
	[26, 37, 38]	The patient (child) was instructed to stand in front of a large screen measuring 200 × 150 cm and played a Kinect-controlled Xbox game that consisted of aerobic exercise (walking and walking with obstacles) games	Depends on the game	20 min
	[39, 40]	A screen-mirror setup was used to induce a walking visual illusion in the patient. The mirror reflected the upper body of the subject, while the screen showed patient-matched legs walking in a treadmill machine The interaction was performed by the mirror part of the setup. Additionally, audio feedback was provided	1PP-mirror	15 min
	[41]	The patient was positioned barefoot in front of a projector on a balance board with which the patient interacts with the VE provided by different games in Nintendo Wii Fit	1PP-mirror and 3PP, depending on game	20 min
Head-based	[34, 35]	The participant underwent two VR experiences, a neutral and a spider VR scene. The interaction consisted of the head position tracking by the HMD	1PP	6 min
	[36]	The participant underwent VR driving scenarios with standardized presentation of 12 warzone events. The interaction consisted in head tracking by the HMD. Combat-related multisensory feedback (visual, auditory, olfactory, and haptic) was provided	1PP	24 min

*N/R* not reported

#### NIBS protocol

In 12 articles, tDCS was reported as NIBS technique, with the most common subtype being anodal tDCS, reported in 10 articles. The duration of the tDCS stimulation was quite consistent, being 20 min the most common duration, reported in 10 articles. The two other tDCS articles reported durations of 13 and 25 min. The current intensity in tDCS was either 1 mA or 2 mA, with 2 mA reported in 8 articles. Four articles made use of TMS, either rTMS or iTBS, the reported durations varied greatly from 3 to 30 min, although the intensity was consistent at 80% or 90% of the resting motor threshold (rMT). Table 5 presents the the NIBS type, subtype, description, duration, and intensity for each of the reviewed articles. The hardware that were used for NIBS in each of the reviewed articles in provided as Additional file 1: Table S1.

**Table 5:** Characteristics of the NIBS protocol

NIBS	Subtype	Article	Description	Duration	Intensity
tDCS	Cathodal	[25]	The cathode was placed over the hand area of the unaffected motor cortex, and the anode over the contralateral orbit of the eye	20 min	2 mA
		[32]	The cathode was placed over the patients’ scalp which corresponded to the primary motor cortex (M1) of the unaffected hemisphere, and the anode over the contralateral orbit of the eye	20 min	2 mA
	Anodal	[30]	The anode placed over motor cortex (M1) in the non-dominant hemisphere in healthy volunteers and the affected hemisphere in stroke patients, and cathode over contralateral supraorbital area	20 min	1 mA
		[31]	The anode was placed over the primary motor cortex (M1), i.e., C3 or C4 (EEG 10–20 system) of the affected hemisphere, and cathode above the contralateral eye orbit	13 min	2 mA
		[33]	The anode is placed over the ipsilesional primary motor cortex (M1), i.e., C3 (EEG 10–20 system), and the cathode is placed in the contralesional supraorbital cortex i.e., Fp2 (EEG 10–20 system)	20 min	2 mA
		[26, 37, 38]	The anodal electrode was positioned over the primary motor cortex contralateral to the lower limb with greater motor impairment, and the cathode was positioned in the supraorbital region on the contralateral side	20 min	1 mA
		[39, 40]	The anode was placed over the motor cortex (M1) contralateral to the more painful hemibody either over C3 or C4 (EEG 10–20 system), and cathode over contralateral supraorbital area	20 min	2 mA
		[41]	The anode was positioned over C1 (EEG 10–20 system) left hemisphere and the cathode was positioned in the supraorbital region contralateral to the anode	20 min	2 mA
		[36]	Anode and cathode were placed over AF3 and PO8 (EEG 10–20 system) respectively. The stimulation aimed the ventromedial prefrontal cortex	25 min	2 mA
TMS	rTMS	[28]	TMS was applied using a 70-mm figure-of-eight air film coil. After defining the motor hotspot and rMT of the contralesional hemisphere, rTMS was applied at a rate of 1 Hz	10 min	90% of rMT
		[29]	TMS was applied using a 70-mm figure-of-eight air film coil. rTMS was applied to the contralesional hemisphere over the primary motor cortex at a rate of 1 Hz	30 min	90% of rMT
	iTBS	[34, 35]	TMS was applied using a 75-mm figure-of-eight air film coil, over the left prefrontal cortex, F3 (EEG 10–20 system) 600 pulses in intermittent biphasic bursts at a frequency of 15 pulses per second via 2 s trains, every 10 s	3 min	80% of rMT

*N/R* not reported

#### VR-NIBS protocol

The use of a combined VR-NIBS protocol for therapeutic applications is quite recent, as consequence, there are not guidelines nor consensus on how to best combine these techniques. Regarding the temporal relationship of VR and NIBS protocols, three different approaches were found: (i) both protocols are administrated simultaneously (reported by 6 studies), (ii) the VR protocol starts (with or without delay) once the NIBS protocol ends (reported by 6 studies), and (iii) the VR protocol is initiated certain period after the NIBS protocol starts, thus there is a partial overlap between them. In all the articles using TMS, the VR protocol started once the stimulation was over, this is due to the mobility limitations of the TMS equipment. This behaviour is less common in studies using tDCS, as tDCS electrodes can be attached to the head, thus it is possible to have mobility and apply tDCS during the VR protocol. In only 2 studies using tDCS, the stimulation was performed before the VR protocol. The temporal relationship between mirror therapy (for stroke rehabilitation) and anodal tDCS was explored in [46]; the evidence showed that the simultaneous use of tDCS and mirror therapy resulted in significant improvement in one motor function test, compared to the use of tDCS before mirror therapy. As such, the simultaneous used of tDCS and (VR) therapy seems to be more advantageous and time efficient. Due to the novelty of the VR-NIBS combination, it is not clear how many sessions of the combined protocol are required for therapeutic applications. A total of 5 articles explored the effects for a single session intervention, while the other studies used a number of sessions that varied between 6 and 25. A description of the temporal relationship between the VR and NIBS protocols, their combined durations and number of sessions are presented in Table 6.

**Table 6:** Temporal relation between VR and NIBS protocols

Therapeutic application	Article	VR duration (min)	NIBS duration (min)	Temporal relation	Total duration (min)	Number of sessions/period
Stroke rehabilitation	[25]	30	20	Simultaneous	30	15 sessions, 5 sessions per week
	[30]	15	20	VR after NIBS end	35	1 session
	[31]	45	13	VR after NIBS end	60	15 sessions, 3 sessions per week
	[29]	30	30	VR started 10 min after NIBS end	N/R	24 sessions, 6 sessions per week
	[28]	N/R	10	VR after NIBS end	N/R	9 sessions, 3 sessions per week
	[33]	20	20	Simultaneous	60	25 sessions
	[32]	20	20	Simultaneous	20	10 sessions, 5 sessions per week
Phobia and PTSD	[34]	6	3	VR after NIBS end	N/R	1 session
	[35]	6	3	VR after NIBS end	N/R	1 session
	[36]	24	25	Simultaneous	N/R	6 sessions in 2 weeks
Cerebral palsy	[37]	20	20	Simultaneous	20	1 session
	[26]	20	20	Simultaneous	20	1 session
	[38]	20	20	Simultaneous	20	10 sessions, 5 sessions per week
Neuropathic pain	[39]	15	20	VR started 5 min after NIBS start	20	10 sessions in 2 weeks
	[40]	15	20	VR started 5 min after NIBS start	20	10 sessions in 2 weeks
Multiple sclerosis	[41]	20	20	Simultaneous	20	5 sessions in 1 week

*N/R* not reported

Lastly, the different combinations of VR system types and NIBS techniques is presented in Table 7. As it can be seen, the combination of tDCS and stationary VR was the most frequently used VR-NIBS protocol. Most of the reviewed articles made use of a stationary VR system. Only three articles reported the use of HMD for the VR protocol, however, with the recent technological advances in computer graphics and sensors, we believe that the use of head-based VR systems will be more common in the near future, as they provide a more immersive experience. Regarding the type of NIBS, tDCS is more practical than TMS, and can be used simultaneously with VR systems.

**Table 7:** Combinations of VR and NIBS protocols

	VR type	
	Stationary	Head-based
NIBS type		
tDCS	[25, 26, 30–33, 37–41]	[36]
TMS	[28, 29]	[34, 35]

### Reported outcomes

The evaluation methods that were used to assess the effects of the VR-NIBS protocol depended on the therapeutic application. In the following subsections, a compilation of the reported evaluation methods and the reported outcomes are discussed for each therapeutic application. Moreover, from the reported outcomes, the overall effect of the VRNIBS protocol is labeled as “positive” or “negative” if there is statistically significant evidence in favor or against the benefits of using the VR-NIBS protocol respectively, or as”neutral” if there is not conclusive evidence reported.

#### Stroke rehabilitation

Stroke rehabilitation was the category with the highest number of reviewed articles likely due to the fact that VR and NIBS techniques have already been widely explored individually. For instance, NIBS has been commonly applied to the ipsilesional sensorimotor and premotor cortex to induce neuroplasticity, thus correlating with recovery [47]. In turn, VR has been widely adopted for a range of motor exercises for neurological rehabilitation [48]. By combining these two techniques, the studies aimed towards increasing excitability within the lesioned hemisphere, either directly through facilitatory stimulation or indirectly through suppressive stimulation to the contralesional hemisphere [28]. To assess the effects of the VR-NIBS protocol, the motor functionality was evaluated with quantitative standard behavioural tests of which the most commonly reported was the Fugl-Meyer scale for upper limb. The reported evaluation methods and outcomes for the stroke rehabilitation application are presented in Table 8. It can be seen that 5 of the 7 articles in this therapeutic application reported positive effects with the use of the VR-NIBS protocol.

**Table 8:** Reported outcomes for articles in the category stroke rehabilitation

Article	Evaluation method	Outcome	Effect
[25]	Evaluation was performed before and immediately after the interventions. The evaluation methods included: modified Ashworth scale (MAS), manual muscle test (MMT), manual function test (MFT), Fugl-Meyer scale (FMS), and box-and-block test (BBT), all these tests to evaluate UL function. Also, Korean-modified Barthel index (K-MBI) was used	Within groups, VR, tDCS and VR-tDCS interventions presented significant improvement in the MMT, MFT, FMS and K-MBI. Between groups, the improvements in MFT and FMS for the VRtDCS intervention were significantly higher than in VR and tDCS interventions	Positive
[30]	Evaluation was performed before, during (the VR part) and after interventions. The corticospinal excitability was evaluated by measuring the changes in amplitudes of motor evoked potentials (MEPs) in the extensor carpi radialis muscle, elicited with single-pulse TMS	VR wrist exercise after tDCS had greater immediate and sustained post-exercise corticospinal facilitation effects than the other interventions. This result was observed in healthy volunteers and subacute stroke patients	Positive
[31]	Evaluation was performed before and immediately after the interventions. The evaluation methods included: FMS, Wolf motor function test (WMFT), MAS, grip strength, and the stroke specific quality of life scale (SSQOL)	VR-sham and VR-tDCS groups showed significant improvements in FMS, WMFT, grip strength and SSQOL. However, in contrast to what was expected, no differences between the groups were observed	Neutral
[29]	Evaluation was performed before, during (each week) and after the interventions. The evaluation methods were the FMS and WMFS	After 4 weeks, participants in the VR-sham and VR-TMS groups presented significant improvement in the FMS and WMFS. The improvement in the VR-TMS group was significantly higher than in the VR-sham group	Positive
[28]	Evaluation was performed before an after interventions. The evaluation methods included the BBT and finger tracking test (FTT). Also the interhemispheric inhibition was evaluated with changes in amplitude of MEPs	Significant motor improvements were observed in both VR-sham and VR-TMS for the FTT, and only the VR-sham showed significant improvement in the BBT. Regarding IHI, it showed significant changes in both groups but in opposite directions, the VR-TMS group showed an increasing ipsilesional fMRI activation during paretic hand tracking	Neutral
[33]	Evaluation was performed before and after the end of each of the three phases. Evaluation methods included: FMS and WMFS	During the VR-tDCS phase, the subject presented an improvement of 86.7% in the FMS, and an improvement of 10.9% and 12% in the WMFS time and ability scores	Positive
[32]	Evaluation was performed before and after the intervention. Clinical assessment included: FMS, the action research arm test (ARAT) and the Barthel Index (BI)	VR-sham and VR-tDCS groups showed significant improvements in FMS, ARAT and BI. Between groups, the improvements in these 3 evaluation methods for the VR-tDCS intervention were significantly higher than in VR-sham intervention	Positive

#### Phobia and PTSD

For these therapeutic applications, the effects of the use of VR-NIBS protocol were measured with self-reported questionnaires, as well as psychophysiological measurements. The study on PTSD, [36], reported a positive effect on self-reported and psychophysiological metrics with the use of VR-tDCS therapy, while the studies with spider phobias [34, 35] reported no effects after VR-TMS therapy, however these both studies only explored the effects of VR-TMS after only one session. The details of the reported evaluation methods and outcomes for this category are presented in Table 9.

**Table 9:** Reported outcomes for articles in the category phobia and PTSD

Article	Evaluation method	Outcome	Effect
[34]	Evaluation was performed at 1 and 3 min after the beginning of the baseline and spyder VR scenes. Evaluation methods included: subjective units discomfort (SUDS), heart rate (HR) and skin conductance level (SCL)	No significant differences were reported between the VR-sham and VR-TMS groups	Neutral
[35]	Evaluation was performed before and after VR challenge. Evaluation methods included fNIRS measurement, during which the participants completed an emotional-word stroop paradigm, also behavior performance (reaction times/error rates) was evaluated	It was not possible confirm a modulatory effect of TMS on either cortical activation, behavioural performance or perceived emotional content of the stimuli	Neutral
[36]	Evaluation was performed at baseline, after each session, and after one month of the intervention. Evaluation methods included psychophysiological arousal (skin conductance reactivity [SCR]) during each VR session, and self-reported PTSD symptoms	Both groups VR-sham and VR-tDCS presented a significant decrease in physiological responding across sessions, this decrease was significantly greater in the VR-tDCS group. Also both groups demonstrated clinically meaningful reduction in PTSD symptoms, but the VR-tDCS group continued improving during the 1-month follow-up	Positive

#### Cerebral palsy

The three studies in this category made use of the same VR-tDCS protocol, with the main difference being the number of sessions administered, namely, one-session protocol was used in [26, 37] and ten-session protocol in [38]. Regarding outcome, the one-session studies in this category reported inconclusive outcomes, while the ten-session protocol reported positive outcomes with the use of the VR-tDCS protocol for children with cerebral palsy. The reported evaluation methods and outcomes for VR-tDCS therapy for cerebral palsy are presented in Table 10.

**Table 10:** Reported outcomes for articles in the category cerebral palsy

Article	Evaluation method	Outcome	Effect
[37]	Evaluation was performed on the same day before and after the intervention. The evaluation method consisted in the timed-up-and-go (TUG) test or placebo) combined with VR training	For the VR-tDCS group there was a within group improvement in the TUG test. However, there was no statistically significant difference between the VR-tDCS and VR-sham groups	Neutral
[26]	Evaluation was performed on the same day before and after the intervention. The displacement of the center of pressure (COP) of the feet in the anteroposterior (AP) and mediolateral (ML) directions was used to analyze body sway in four conditions: eyes open or closed with ground or foam mat as support base	From the analysis of body sway, for the VR-sham group sway velocity in ML direction was significantly different with the foam mat support for eyes closed and open, and for ground support with eyes open. For the VR-tDCS group sway velocity in ML direction was significantly different with the foam mat support for eyes closed and open, and sway velocities in AP and ML directions were significant for the ground support for both eye conditions. No significant differences were reported for between VR-sham and VR-tDCS groups	Neutral
[38]	Evaluation was performed before, immediately after, and one-month after the intervention. The evaluation methods included: gait analysis, the gross motor function measure (dimensions D and E), the pediatric evaluation disability inventory (PEDI) and the motor cortex excitability measured through the amplitude of MEPs	Gait velocity showed significant improvement in the post-treatment evaluation for both VR-sham and VR-tDCS groups. While gait cadence was only significant improved in the VR-tDCS group. Analysis between groups showed that the improvements in gait velocity and cadence were significantly better in the VR-tDCS group. This significant improvement was observed for the gross motor function measures. There were also significant changes in the MEP amplitudes for the VR-tDCS group, but not for the VR-sham group	Positive

#### Neuropathic pain

The two studies in this category made use of numeric rating scales to evaluate the pain intensity. Also changes in the contact heat-evoked potentials were used to assess the effects of the VR-tDCS therapy. Both articles reported positive effects with the combined use of VR and tDCS in neuropathic pain in patients with spinal cord injury. The VR-tDCS protocol used in each of these two studies was applied in 10 sessions during 2 weeks. Reported evaluation methods and outcomes for this category are presented in Table 11

**Table 11:** Reported outcomes for articles in the category neuropathic pain

Article	Evaluation method	Outcome	Effect
[39]	Evaluation was performed before, after the last day of intervention, after 2, 4 and 12 weeks for follow-up. The evaluation methods included numeric rating scales (NRS) for pain intensity, interference with function, anxiety	The VR-tDCS intervention significantly reduced the intensity of neuropathic pain, more than the VR, tDCS and placebo interventions	Positive
[40]	Evaluation was performed before and immediately after the intervention. Evaluation methods included: NRS for NP intensity, study of warm and heat pain threshold, recording of contact heatevoked potentials (CHEPs) to thermal stimulation, and NRS for CHEPs evoked pain perception	Two weeks of VR-tDCS induced significant changes in CHEPs, evoked pain and heat pain threshold in SCI patients with NP. Thirteen patients reported a mean decrease of 50% in the NRS for NP after VR-tDCS	Positive

#### Multiple sclerosis

For this category, there was only one study which involved only one participant. For sake of consistency with the previous subsections, the reported evaluation methods and outcomes for this study are presented in Table 12.

**Table 12:** Reported outcomes for articles in the category multiple sclerosis

Article	Evaluation method	Outcome	Effect
[41]	Evaluation was performed before, after and 14 days after each intervention. Evaluation methods included: the balance evaluation systems tests (BESTest), the modified fatigue impact scale (MFIS) and the functional determination scale of quality of life for MS	No differences were found between VR with active and VR with sham tDCS interventions in relation to balance, fatigue, and quality of life	Neutral

In summary, across the different therapeutic applications, 9 of the 16 reviewed articles reported positive effects regarding the combined use of VR and NIBS. The remaining studies reported neutral or inconclusive outcomes regarding the use of VR-NIBS therapy, and no article reported negative effects. These outcomes suggest that VR and NIBS techniques might successfully complement each other. More studies are needed to define specific VR and NIBS protocols for each therapeutic application, and to corroborate the reported findings in larger populations. Four of the seven articles with neutral effects, [26, 34, 35, 37], used a single-session approach for the VR-NIBS therapy. For the four studies using the VR-TMS protocol, only [29] reported positive outcomes with a TMS protocol of 30 min in 24 sessions, while the other three studies used a TMS-protocol for 10 or less minutes in 1 or 10 sessions.

### Reported limitations

Most of the reviewed articles reported one or more limitations from the conducted studies. The most frequently reported limitations were the small sample size, and the use of single-session protocols. In the case of stroke rehabilitation studies, a common reported limitation was the heterogeneity in type of lesion, this can have a large impact in the assessment of the VR-NIBS therapy as subcortical stroke patients with intact cortical connectivity may profit more from tDCS than patients with disrupted neural pathways due to cortical stroke [30]. Table 13 presents the reported limitations for each of the therapeutic applications. In studies using TMS, a limitation was the complexity of the study design as the characteristics of the TMS devices difficult other measurements, this is not always the case with tDCS. For example, in all the studies reporting the simultaneous use of VR and NIBS, none reported TMS a NIBS technique.

**Table 13:** Reported limitations

Therapeutic application	Article	Limitations
Stroke rehabilitation	[25]	(1) Small number of enrolled patients. (2) There was no comparison between cortical and subcortical lesions
	[30]	(1) Small sample of mildly impaired stroke patients. (2) All subacute stroke patients were in a period of spontaneous recovery. (3) There was no comparison between cortical and subcortical lesions. (4) Lack of sham stimulation or multiple mode simulation of tDCS
	[31]	The small sample size could have influenced the absence of group differences, since the sample size is related to the power analysis
	[29]	(1) It was not possible to obtain solid evidence for any functional change in the brain using functional MRI or PET. (2) Most patients had spontaneous recovery of motor function. (3) The small sample size, lack of multiple center involvement, and short-term evaluation and follow-up were factors increasing the ambiguity in terms of long-term therapeutic effect and experiment consistency
	[28]	(1) The small sample size.(2) There was no control condition.(3) Difficulty at identifying eligible subjects
	[33]	Future studies are needed to determine whether the observed changes were promoted by the intervention itself or by a change of intervention
	[32]	(1) Small sample. (2) Only single-blinded. (3) Patients have additional rehabilitation therapies in the medical center
Phobia and PTSD	[34]	(1) There was only one NIBS session. (2) Although the study was successfully blinded, some participants reported sensations during active iTBS. (3) Baseline measurements of HR, HRV and SCL were recorded after iTBS only due to the complexity of study design
	[35]	(1) The delay (due to the study design) between the use of iTBS and the measurement with fNIRS may attenuate the effects of iTBS. (2) The use of iTBS may had induced counteracting effects: better cognitive control (thus better emotion regulation), and diminished feeling of presence in the VE
	[36]	The small sample size
Cerebral palsy	[37]	N/R
	[26]	N/R
	[38]	(1) The small sample size. (2) The lack of exploration of different electrode positions and tDCS protocols
Neuropathic pain	[39]	N/R
	[40]	(1) There was no control condition for the tDCS-VR intervention group. (2) The study was not blinded for patients. (3) The intervention always included both tDCS and VR, so that it was not possible to discriminate between the effects on pain of each separately
Multiple sclerosis	[41]	(1) Sample size. (2) Patient reported itching sensation after VR-tDCS session

*N/R* not reported

## Recommendations

As it has been highlighted in numerous studies, there are several issues regarding combining VR and NIBS that still need to be addressed. In this section, we compile these issues as recommendations for future studies. Regarding the reported VR protocol, we found that important details about it are often disregarded, thus limiting reproducibility, e.g., the studies where the VR-protocol was based on videogame consoles, such as Nintendo Wii or Xbox, ( [26, 31, 37, 38]), it was not reported the specific games and mini-games that the participants experimented. This information is crucial, as for a given title, there are mini-games with 1PP and 3PP viewpoint. It is thus recommended that future studies report as much detail about the VR protocol as possible. Next, regarding the way that VR and NIBS were combined, their temporal application is not often fully described. For example, the duration of the pauses between techniques (if any) are rarely reported. It is recommended that improved timing details be provided in future studies. Regarding the impact of the number of sessions, based on outcomes reported in “Reported outcomes” section, only one, [30], of the five articles reporting a single session of VR-NIBS reported positive outcomes. Moreover, although the articles [26, 37] and [38] reported the same VR-tDCS protocol, neutral outcomes were reported in [26, 37] after one session, and positive outcomes were reported by [38] after 10 sessions. As such, evidence suggests that the VR-NIBS protocols may not have an effect in one-session interventions. As VR-NIBS protocols are still a novel and under explored, there are several aspects that future research still needs to address. Indeed, none of the articles here reviewed explored the effects of characteristics such as number of sessions, temporal relationship, or duration of the VR-NIBS protocol performance. As such, it is recommended that future studies focus on aspects such as the optimal duration of NIBS, and standardized metrics to measure outcome performances. Lastly, to allow comparisons with former studies, we recommend future authors in the field to use Table 1, as a checklist of the information that must be present when reporting their results.

## Conclusions

The present review has explored the published findings related to the effects and reported outcomes of the combined use of VR and NIBS in therapeutic applications. While the reported outcomes suggest that the combination of VR and NIBS has a great potential in different therapies (e.g., stroke rehabilitation, cerebral palsy, phobias, PTSD, and neuropathic pain), many limitations still exist. In particular, additional evidence must be acquired, and studies need to better describe the experimental protocols in order to foster study replication. We believe the reported findings as well as the recommendations provided in this review may help future researchers to better understand VR and NIBS techniques and develop better protocols for their combined use.

## Supplementary information

**Additional file 1: Table S1.** Hardware and software used for the VR and NIBS protocols.

## Data Availability

All data generated or analysed during this study are included in this published article.

## Abbreviations

1PP: First-person perspective
3PP: Third-person perspective
BCI: Brain-computer interface
CHEP: Contact heat evoked potentials
EDA: Electrodermal activity
EEG: Electroencephalogram
FMS: Fugl-Meyer scale
HMD: Head mounted displays
HR: Heart rate
HRV: Heart rate variability
IMI: Intrinsic motivation inventory
MAS: Modified Ashworth scale
MBI: Korean-modified Barthel index
MFT: Manual function test
MMT: Manual muscle test
NIBS: Non-invasive brain stimulation
NP: Neuropathic pain
NRS: Numerical rating scale
PET: Positron emission tomography
PFC: Prefrontal cortex
PTSD: Post-traumatic stress disorder
SCI: Spinal cord injury
SSQOL: Stroke specific quality of life scale
SUS: System usability scale
TMS: Transcranial magnetic stimulation
TUG: Timed Up and go
UL: Upper limb
VE: Virtual environment
VMPFC: Ventromedial prefrontal cortex
VR: Virtual reality
WMFT: Wolf motor function test
fMRI: Functional magnetic resonance imaging
fNIRS: Functional near Infrared Spectroscopy
iTBS: Intermittent theta burst stimulation
rMT: Resting motor threshold
rTMS: Repetitive transcranial magnetic stimulation
tACS: Transcranial alternating current stimulation
tDCS: Transcranial direct current stimulation
tES: Transcranial electrical stimulation
tRNS: Transcranial random noise stimulation
