# Supplementary material for: Virtual reality and non-invasive brain stimulation for rehabilitation applications: a systematic review
Source: J Neuroeng Rehabil. 2020 Oct 31;17:147. doi: 10.1186/s12984-020-00780-5 (PMC7603766; doi:10.1186/s12984-020-00780-5)
Supplement: Supplementary file 1 — Additional file 1: Table S1. Hardware and software used for the VR and NIBS protocols. [file 12984_2020_780_MOESM1_ESM.docx]

# Additional file

Table S1: Hardware and software used for the VR and NIBS protocols.

| **Article** | **VR** | | **NIBS** |
| --- | --- | --- | --- |
|  | **Hardware** | **Software** | **Hardware** |
| [25] | Immersive rehabilitation exercise (IREX), GestureTek (Canada). | Three VR applications: ”Birds and Balls”, ”Conveyor”, and ”Juggler”. Applications developed by GestureTek (Canada). | tDCS Phoresor II Auto Model PM850, ioMED (USA). |
| [30] | Monitor, camera-based hand movement tracker Webcam pro 9000 Logitech Inc (Switzerland), and a cylinder-like interface. | Ski game for wrist exercise following stroke, Metasio Asia Inc.  (Korea). | tDCS Phoresor II Auto Model PM850, ioMED (USA). |
| [31] | Nintendo Wii and controller to detect user’s physical motions. | Three Nintendo Wii titles: “Wii Sports resort”, “Wii Play Motion”, and “Let’s Tap”. | tDCS Striat, IBRAMED  (Brazil). |
| [29] | BioMaster system, Jumho Electric Co.(China) comprised of wearable data gloves with sensors. | VR application for BioMaster system, Jumho Electric Co.  (China). | TMS Magstim Rapid2 stimulator, Magstim Company, (UK). |
| [28] | Stimulus box, and angular mounted screen for 3D VR stimulus. | N/R | Magstim Rapid2 stimulator, Magstim Company, (UK). |
| [33] | Laptop computer, armband Myo, Thalmic Labs (Canada) to acquired arm EMG, position and provide haptics, and eye-tracker EyeX, Tobii Technology AB  (Sweden). | Own developed software in  Unity. | EEG/tDCS headset, the  StarStim 8, Neuroelectrics  (Spain). |
| [32] | VR system made by Shanghai Fourier Intelligent Technology Co., Ltd. (China). Consisted of Feedback sensing manipulator and a large screen. | VR application by Shanghai Fourier Intelligent Technology Co., Ltd. (China). | tDCS IS300 by Sichuan Intelligent Company (China) |
| [37, 26, 38] | Xbox and kinect, and a large screen (2 m x 1.5 m). | Xbox title: ”Your Shape: Fitness Evolved 2012”. | tDCS Soterix Medical Inc.  (USA). |
| [39,  40] | Vertical mirror (1.5 m x 0.5 m), and projector. | Video showing the patient matched legs. | DC-Stimulator by neuroConnGmbH (Germany). |
| [41] | Nintendo Wii and Wii Fit board/platform. Projector, screen size and distance were not reported. | Nintento Wii Fit games: ”Marble Balance”, ”Ski Slalom”, and ”Tight Hope Walk”. | DC-Stimulator Plus,  neuroConn-GmbH (Germany) |
| [34,  35] | Z800 3D HMD (800 x 600 pixels) eMagin (USA), Patriot electromagnetic tracking device (Polhemus Corporation (USA). | VE created with Source Engine, Valve Corporation (USA), simulation controlled by CyberSession software (Germany). | MagOption/MagPro X100 stimulator, MagVenture, (Denmark). |
| [36] | HMD (Sony HMZ-T3), head tracker, devices for olfactory and haptic feedback are not described. Hardware integrated by  Virtually Better Inc (USA). | Three driving scenarios with 12 warzone events. VR application Bravemind by Virtually Better Inc. (USA). | DC-Stimulator Plus,  neuroConn-GmbH (Germany) |
